# Rethinking chronic pain: a dissociative framework for psychodynamic practice

**DOI:** 10.3389/fpsyg.2025.1690309

**Published:** 2025-10-27

**Authors:** Tim Ho, Mark Ryan, Skye Dong

**Affiliations:** ^1^Sydney Medical School, Westmead Clinical School, Faculty of Medicine and Health, The University of Sydney, Sydney, NSW, Australia; ^2^Cingulum Health, Sydney, NSW, Australia; ^3^Birchtree Centre, Sydney, NSW, Australia

**Keywords:** chronic pain, dissociation, psychodynamic, embodiment, trauma

## Abstract

Chronic pain is increasingly recognised not only as a disorder of sensory processing but also as a disruption of self-experience. This article advances the concept of *stimulus entrapment* as a dissociative analogue in chronic pain, where consciousness becomes trapped in overwhelming bodily sensation, narrowing attention and preventing integration into coherent narrative or identity. This entrapment parallels dissociative states in trauma, where overwhelming affect fragments continuity of self. We argue that in chronic pain the “pain self” is often rejected and disowned, creating a split between the embodied, suffering self and the socially presented self. Such fragmentation amplifies distress, sustains alienation from the body, and fosters maladaptive coping. Conceptually, this framework links chronic pain to psychodynamic and dissociation theories, situating persistent pain as a disorder of disrupted self-integration. Framing chronic pain in this way clarifies why biomedical interventions alone often fail to restore function, since the problem lies not only in nociceptive signalling but also in fragmentation of selfhood. Psychodynamic approaches that emphasise narrative repair, emotional attunement, and restoration of self-coherence may reintegrate the “pain self” into the broader self-system, reduce alienation, and restore agency. We propose that stimulus entrapment offers a unifying conceptual bridge between chronic pain, trauma, and dissociation, opening new avenues for therapeutic engagement and future research.

## Introduction

Psychotherapy for chronic pain has traditionally centred on cognitive-behavioural therapy (CBT), a structured approach that targets maladaptive thoughts and behaviours to enhance self-regulation and interrupt the cycle of pain and distress. While meta-analyses show CBT and related psychological therapies reduce pain and distress compared with no treatment or usual care, the effect sizes are often small and long-term functional gains modest (Eccleston et al., 2008; Ho et al., 2022; Leung et al., 2025). Importantly, CBT presupposes a baseline of cognitive and emotional capacity that may be compromised in many individuals with chronic pain, particularly those affected by complex trauma, emotional dysregulation, and prolonged suffering (Ehde et al., 2014).

Psychodynamic therapy offers an alternative, relational model that addresses these limitations. It emphasises right-hemisphere processes—emotional tone, metaphor, non-linear meaning—while integrating left-hemisphere functions such as language, reflection, and logical thought. In the Conversational Model, Meares highlights how right-hemisphere engagement through prosody, gaze, and shared presence mirrors early caregiver–infant protoconversation, fostering relational attunement and a cohesive sense of self (Meares, 2005; Meares, 2012a).

Trauma is highly prevalent in chronic pain populations, not only as an initiating factor (e.g., injury) but as an ongoing relationally mediated experience. Trauma types most associated with dissociation and self-fragmentation—severe and prolonged childhood trauma, early relational trauma, abuse, chronic invalidation—are frequently observed in these cohorts (Bedard-Gilligan and Zoellner, 2012). Moreover, emotional and physical pain share neural substrates in regions such as the anterior cingulate cortex and insula, suggesting overlapping mechanisms for disruption of selfhood. Persistent, unrelieved pain, compounded by healthcare invalidation, social isolation, and identity loss, contributes to what van der Kolk (2014) terms allostatic load: the cumulative stress burden that overwhelms integrative capacity. Over time, this fragmentation of self disrupts narrative coherence and fosters dysregulation and dissociation (Gasperi et al., 2021). At its core, psychodynamic therapy aims to restore self-coherence. This raises a key question: can chronic pain itself involve fragmentation of the self?

This paper therefore proposes that chronic pain, in some cases, reflects a dissociative process: a fragmentation of embodied selfhood arising from cumulative trauma and emotional overload. Psychodynamic therapy may be particularly suited to address this process by restoring self-cohesion, reactivating symbolic and reflective functions, and providing a relational space where fragmented experiences can be named, symbolised, and integrated into a coherent, emotionally meaningful narrative.

## Case description

Nancy, a 50-year-old woman, presented with severe, treatment-refractory low back and leg pain persisting for 4 years after a workplace lifting injury. The injury caused a lumbar disc protrusion, radiculopathy, and failed spinal surgery syndrome, leaving her with disabling neuropathic pain unresponsive to multiple interventions. She describes the surgery as having “made everything worse,” encapsulating both medical failure and her sense of betrayal by care.

Her distress extends well beyond the physical. She feels abandoned by health and compensation systems, dismissed by clinicians, and coerced into premature return to work. Once grounded in roles as mother, carer, and worker, she now describes herself as “broken” and “nothing—just pain.” Episodes of rage, such as “smashing dishes,” erupt in her family environment, followed by shame and withdrawal—expressions of unintegrated affect that overwhelm her regulatory capacity.

**Figure 1:**
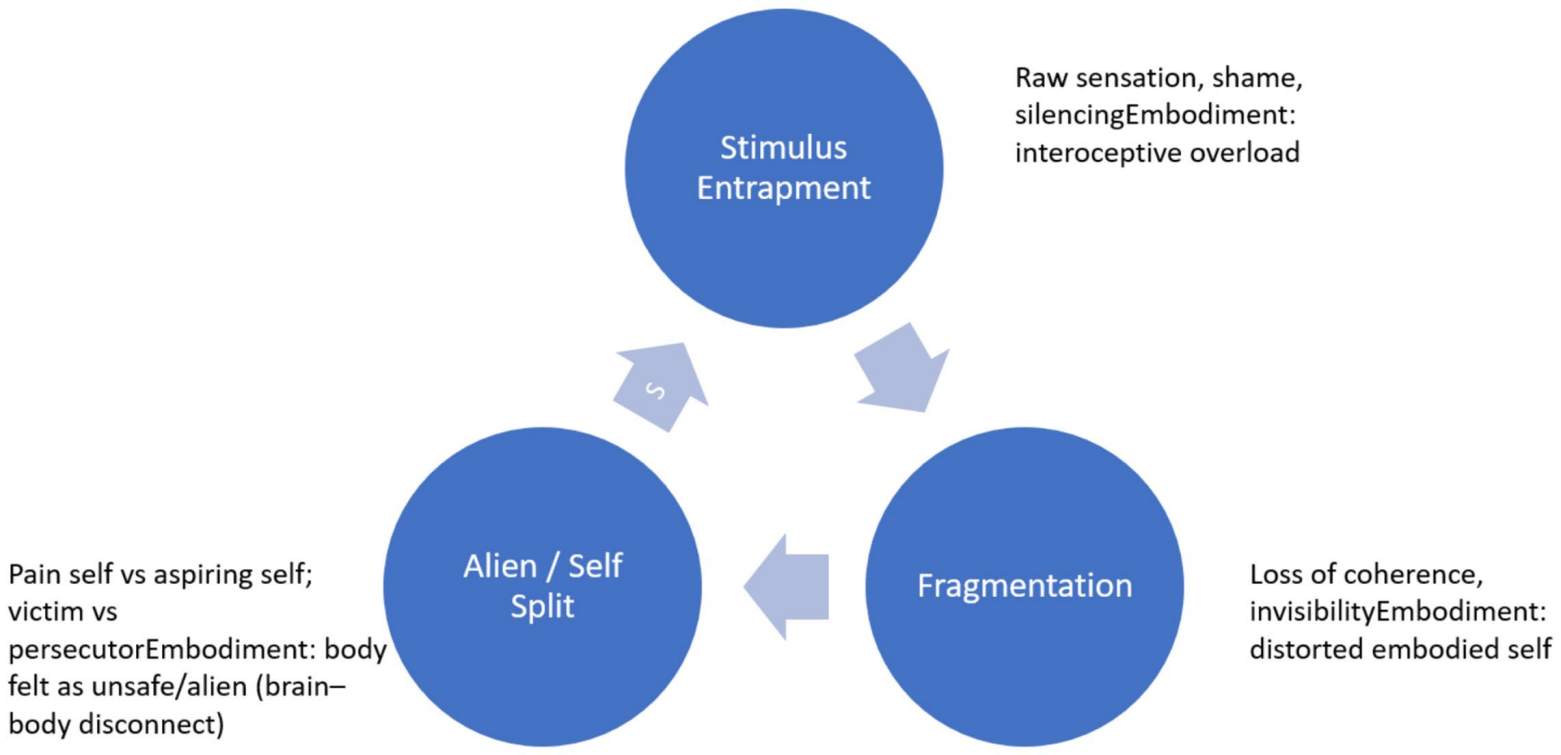
This figure illustrates chronic pain as a dissociative cycle comprising stimulus entrapment, fragmentation, and alienation/self-split, which reinforce one another in a vicious loop. At each stage, trauma disrupts embodiment: bodily signals are overloaded, distorted, or experienced as alien, leading to disconnection from the self. Therapeutic repair offers an exit pathway, where relational safety and interoceptive awareness enable re-integration of the embodied self.

In therapy, Nancy communicates in a flattened, monotone style, listing symptoms and events with little emotional inflection. She averts her gaze, shifts topics abruptly, and appears disengaged from the relational field, as if speaking into emptiness. This presentation aligns with Meares’s concept of *stimulus entrapment*—consciousness trapped in raw bodily sensation without access to narrative or symbolic meaning. Her body becomes both focus and prison of her selfhood: hyper-present in pain yet absent as a source of agency. The therapist may feel drawn into a parallel deadness, echoing countertransference reactions seen in somatization states. In this paper, we use somatization states interchangeably with somatoform dissociation, the latter being associated with worsened trans-diagnostic outcomes in the treatment of PTSD symptoms (Wainipitapong et al., 2025; Kratzer et al., 2021). The somatoform dissociative continuum have been proposed to traverse three degrees of symbolicity: the metaphorical, the metonymic, and the psychotic (Amir, 2023) (see Figure 1).

Nancy’s difficulties reflect the convergence of several factors. Early vulnerabilities likely included attachment disruptions where emotional needs were unmet, leaving somatic expression as a primary channel of communication. The workplace injury and failed surgery served as precipitants, re-enacting themes of abandonment, invalidation, and betrayal. Perpetuating influences include ongoing neuropathic pain, entrenched dissociative coping, relational ruptures from uncontrolled affect, and preoccupation with symptoms that narrow inner life. Protective factors remain: she is articulate, engaged in therapy, and retains a desire for connection, albeit fractured.

Nancy’s case illustrates how chronic pain can operate both as symptom and as dissociative language. The therapeutic task is not only pain management but the restoration of embodied self-in-relationship. Attunement to embodied intersubjectivity—subtle shifts in rhythm, vitality, and affect—offers a pathway to reconnect fragmented experience. By meeting her cry of “I’m nothing, just pain” as an intersubjective signal rather than literal truth, therapy can transform pain from an isolating object into part of a shared narrative of recovery and selfhood. Qualitative research supports this view: patients frequently describe pain as alien, persecutory, or personified, consistent with dissociation of the “pain self” (Tsur, 2020). Unlike somatization, which conveys distress through bodily expression, *somatoform dissociation* reflects disconnection from bodily signals, experienced as alien or uncontrollable—a disruption that links trauma, pain, and dissociation (Bob et al., 2013).

## On dissociation

Dissociation refers to a disruption in the continuity of self-experience—an adaptive response to overwhelming affect or trauma. The DSM-5 defines it as a “marked discontinuity in the sense of self, sense of agency, and related alterations in affect, behaviour, consciousness, memory, perception, cognition, and/or sensorimotor functioning” (American Psychiatric Publishing, 2015). Janet (1907) described this as a “failure of personal synthesis,” highlighting a breakdown in the integration of subjective experience.

Patients often describe dissociation in simple experiential terms: “I do not feel real,” or “I’m watching myself from outside.” It exists along a spectrum—from transient, everyday lapses such as daydreaming to severe, pathological states that fragment identity and impair functioning (Holmes et al., 2005). At the extreme, Dissociative Identity Disorder (DID) demonstrates how the mind can split into distinct personality states, often separated by amnesia, usually linked to severe and chronic childhood trauma, particularly emotional, physical, or sexual abuse (Boyer et al., 2022). While chronic pain rarely involves DID, it may reflect milder but still clinically significant dissociative states—underscoring the continuum of self-fragmentation in trauma-affected individuals.

Meares (2012b) offers a developmental model of dissociation, consistent with evidence that dissociation and somatic symptoms often cluster in trauma populations (Kratzer et al., 2021):

- *Primary dissociation* arises when early trauma or disorganised attachment disrupts coherent self-formation, leaving experiences unintegrated and inaccessible to narrative memory.
- *Secondary dissociation* involves compartmentalisation under overwhelming stress, producing numbing, shutdown, and narrowed awareness. Protective in the moment, it impairs reflection and integration.
- *Tertiary dissociation* reflects chronic fragmentation, where identity splits into disconnected parts, generating pervasive incoherence (Wilkinson-Ryan and Westen, 2000).

Functional Neurological Disorder (FND) provides another window onto dissociation. FND manifests as neurological symptoms such as seizures or paralysis without identifiable pathology. Research suggests these symptoms reflect a dissociative process: trauma and emotional distress that remain unprocessed, unintegrated, and therefore expressed somatically (Meares, 2012a; Campbell et al., 2022). When narrative and symbolic expression fail, excessive stress severs mind from body. Chronic pain, like FND, can also be understood as a somatic expression of dissociation, particularly when trauma remains unresolved.

Taken together, dissociation can be understood as a layered, adaptive spectrum—from everyday detachment, through compartmentalisation and identity fragmentation, to bodily manifestations such as FND. The stream of self may fragment across cognition, emotion, bodily sensation, or perception—each a facet of dissociation’s protective but ultimately isolating impact.

## Dissociation as trauma to the self

The self is not fixed but a dynamic, relationally constructed process—an interwoven system of bodily sensations, affective responses, relational templates, memory networks, and narrative continuity (Siegel, 2015). From early development, the self emerges through attuned interactions: play and emotional mirroring within caregiving relationships foster the capacity to symbolise, reflect, co-create, and integrate inner experience (Guthrie and Moorey, 2018; Meares, 2005). Fuchs and Koch (2014) describe affectivity as inherently bodily and relational, captured in the phrase “moving and being moved”. Through such interactions, thoughts, feelings, and bodily states cohere into a fluid narrative of “I am.” Kohut (2009) similarly framed the self as an emergent, relational phenomenon shaped by being seen, mirrored, and validated.

Fonagy et al. (2018) emphasised the role of inner privacy—the capacity to hold one’s own thoughts and feelings—which aligns with Meares’s metaphor of play (Meares, 2005), where imaginative exchange fosters symbolic thought and selfhood. This symbolic function underpins intimacy, empathy, and identity, allowing the self to integrate the “I” (the knower) with the “Me” (the known) through narrative and metaphor. Legrand (2007) makes a similar distinction between the pre-reflective self-as-subject and the reflective self-as-object.

Cay et al. (2022) demonstrated that childhood maltreatment predicts chronic pain and psychiatric comorbidity, underscoring trauma’s role in pain-related self-fragmentation. Recent studies confirm that trauma distorts embodiment and disrupts the integration of bodily signals into coherent identity, with childhood abuse amplifying somatic fear responses (Ong et al., 2025). Ong et al. (2025) extend this by showing how childhood abuse alters the embodied sense of self, mediating later distress and somatic fear responses. In Borderline Personality Disorder, such disruption manifests as an unstable, incoherent self—experienced as alien, shifting, and painfully fragmented (Lanius et al., 2020).

From a neurobiological perspective, Meares (2012b) situates self-coherence within the Default Mode Network (DMN), which underpins autobiographical memory, introspection, and the sense of self. Trauma disrupts this system: overwhelming stress or relational failure (e.g., neglect, coercion) fragments the layered organisation of sensory, emotional, and cognitive processes, resulting in a breakdown of self-integration (Meares, 2012a). This may help explain why interventions targeting core DMN functions—particularly interoceptive approaches—have shown promise in restoring embodied self-coherence in PTSD and chronic pain (Putica et al., 2025; Putica, 2025).

From an evolutionary perspective, dissociation functions as a survival adaptation that fragments embodied and relational processes (Burback et al., 2024). Symbolically, dissociation can be seen as a restriction of the stream of consciousness—a survival response when the flow of emotion, memory, and identity becomes unbearable. What cannot be integrated is split off, left behind as a stagnant pool: the lost self, the muted emotion, the silent memory. Cut off from the living current, these “dead waters” remain unfelt and unspoken, leaving the self disconnected, the story incomplete, and the heart yearning for reconnection (van der Kolk, 2014; Siegel, 2015).

## Chronic pain as a dissociative state

Chronic pain is not merely a continuation of acute injury but a persistent, unresolved stressor that overwhelms the self’s integrative capacity. Defined as pain lasting longer than 3 months, it constitutes an *allostatic load*—sustained physiological, psychological, and social stress that depletes the system’s ability to regulate and recover (Liang and Booker, 2024). The soma, once embodying safety and agency, may, under the grip of chronic pain–induced stimulus entrapment, be experienced as alien and threatening. Kearney and Lanius (2022) describe this as a trauma-related brain–body disconnect, where sensory and interoceptive signals are experienced as unsafe or foreign. Like trauma, chronic pain is an experience of threat without escape, effort without restoration, leading to breakdowns in coherence of self. The “pain self” is often disowned and split off, mirroring dissociation in which overwhelming experience is rejected rather than integrated.

Relational trauma may further compound this collapse. Patients with chronic pain frequently encounter dismissal, disbelief, and invalidation from healthcare systems, insurers, and even family (Scarry, 1985). Most corrosive is *betrayal trauma* (Freyd, 1994), where trusted systems become sources of harm, reactivating attachment wounds and deepening alienation. Disorganised attachment—commonly linked to early relational trauma—may further perpetuate pain, amplifying emotional complexity and leaving individuals feeling trapped and fragmented.

The consequences for selfhood are profound. Loss of identity reflects erosion of social roles and purpose—the carer, the worker, the parent—the erosion of the “Who am I?” story. Self-fragmentation goes deeper, reflecting breakdown of the integrative processes that sustain continuity across body, mind, emotion, and time (Meares, 2000). Unlike minimisation or intellectualisation, which attempt to control distress, dissociation represents a psychic retreat: an absence of emotional presence despite outward engagement (Allen et al., 1999; Slonim, 2014).

Clinically, dissociation in chronic pain manifests as compartmentalisation of affect, memory, and agency. Survivors may even personify pain as an external persecutor, consistent with pain functioning as a dissociative self-part (Tsur, 2020). Common expressions include:

- *Fragmented identity*: “I used to be a nurse, a mother, a runner… now I’m just pain.”
- *Disconnection from the body*: “It’s like my leg does not belong to me.”
- *Affective numbing*: “I do not have depression—it’s just pain.”/“It’s not in my head.”
- *Narrative collapse*: Stories of pain delivered as factual, stripped of emotional tone or metaphor (Scarry, 1985).

Nancy’s case illustrates these dynamics. Following injury, failed surgery, and systemic invalidation, she described herself as “broken… nothing… just pain.” Her narrative was flattened, affect muted, and self fragmented. Outbursts such as “smashing dishes” emerged as unintegrated fragments of experience—a dissociative compromise (van der Kolk, 2014) that shielded her from overwhelming stress. These eruptions may signify not only dysregulation arising from a disrupted self but also reactivation of trauma. Tsur and Katz (2023) identified *pain flashbacks* as intrusive trauma symptoms. In some cases, this takes the form of *reversal*, with roles of victim and perpetrator inverted, or *eruption*, where traumatic fragments resurface somatically or affectively rather than verbally.

Older constructs such as alexithymia, somatization, and catastrophization describe observable features of disrupted processing of chronic pain. A dissociative perspective reframes these not as separate syndromes but as downstream expressions of a deeper rupture in self-integration. The “pain self” can thus be understood as a dissociative part organised around embodied suffering.

Emerging evidence supports this link: trauma-related intrusions, peritraumatic dissociation, and failures of emotional processing frequently manifest somatically. Chronic pain may operate as a re-experiencing of trauma, rooted in early adversity and PTSD (Danböck et al., 2023; Hellman et al., 2025; Hettegger et al., 2025; Karimov-Zwienenberg et al., 2024; Tsur and Katz, 2023). Hellman et al. (2025) similarly argue for integrating chronic pain within trauma-processing models, reinforcing dissociation as a central mechanism.

## Managing dissociation in chronic pain

If chronic pain reflects a dissociative process—an adaptive survival response to overwhelming trauma—then healing requires repair of the fragmented self and reconnection with body, mind, and emotion (Meares, 2012a).

Psychodynamic therapy offers a relational space where dissociation can be recognised, named, and gently explored. Dissociation is not treated as resistance but as a silent expression of unspoken trauma and a call for presence (Meares, 2005). While long-term therapy is needed to rebuild the self, brief therapy can reawaken capacities for connection, emotional resonance, and narrative flow, develop therapeutic appliance, address challenging comorbidities, and establish a strong foundation for trauma and integration work to follow (Kluft, 2023; Kluft, 2022). Even limited experiences of secure attachment and emotional exploration can seed growth, allowing patients to feel seen, felt, and heard.

Recognising dissociation requires relational attunement—sensitivity to subtle cues of disconnection, fragmentation, and unspoken suffering, and attention to the *minute particulars* that reveal a self in hiding (Meares, 2005; Guthrie et al., 2001). Working with dissociation demands sensitivity to subtle shifts in the therapeutic field: gaze aversion, flattened tone, abrupt topic changes, or a sense that the patient has “gone far away.” Such moments can be gently acknowledged:

*“It feels like something just shifted—like part of you stepped back. I wonder if something is feeling too much or hard to say?”*

Naming these shifts anchors them in the relational space, while the therapist’s regulating tone, slowed pacing, and emotional presence provide containment (Meares, 2005).

Rather than insisting on linear narrative, the therapist invites exploration of the felt sense through metaphor, imagery, or sensation. Patients may describe numbness as “like being underwater” or disconnection as “I feel invisible.” These symbolic expressions give voice to dissociated states, bridging implicit and explicit memory, and integrating bodily sensation, emotion, and cognition within a single image (Fosha, 2000).

In Nancy’s therapy, dissociation appeared as monotone recounting of medical events, gaze aversion, and a sense that her body was no longer hers. The therapeutic focus was to soften these moments, inviting her to slow down, notice feelings, and symbolise experience:

*“If this pain were a monster, what would it look like?”*

Through such non-linear, emotionally attuned exchanges—what Meares (2000) calls the *poetic voice*—her fragmented experiences surfaced safely and were woven into a more coherent narrative.

Healing in psychodynamics is not about “fixing” dissociation but restoring the capacity for play, emotional connection, and co-regulation (Meares, 2012a; Winnicott, 1991). By slowing the pace, softening language, and creating pauses in Nancy’s repetitive, list-like accounts, therapy opened portals into deeper relational themes—fear of rejection, feeling unseen, or disappearing to stay safe. Winnicott and Meares remind us that recovery comes not from insight alone but from rediscovering shared rhythms—pauses, silences, attuned presence—where the hidden self feels safe enough to emerge. Therapy becomes a dance: a fluid rhythm where the self can step forward, not to erase the past, but to reclaim its place in life and co-create a new music of connection.

## Limitations

This article is conceptual in nature and does not present empirical data. While grounded in clinical observation, psychodynamic theory, and existing research, the proposed model of chronic pain as a dissociative process remains hypothetical. Its validity and clinical utility should be examined in future studies using longitudinal, neurobiological, and qualitative methodologies. Further studies on interventions targeting the trauma sequelae should address individual symptom profiles of each patient and use a dynamic and trans-diagnostic approach to treatment (Kratzer et al., 2021).

## Conclusion

This paper reframes chronic pain as a dissociative disorder—an adaptive fragmentation of self in response to trauma, relational betrayal, and allostatic overload. Psychodynamic therapy offers a relational, attuned approach that fosters re-integration through emotional connection, metaphor, and shared narrative. While empirical evidence for psychodynamic therapy in chronic pain remains limited, this model underscores the therapeutic potential of recognising dissociation as a call for relational healing of the self. We propose that chronic pain can be understood as a dissociative phenomenon, arising through stimulus entrapment that leads to fragmentation and alienation of the self. Clinically, we hypothesise that therapies aimed at restoring embodied self-coherence—such as psychodynamic and interoceptive approaches—may help reintegrate the “pain self” and reduce suffering. Future research should empirically test these propositions to clarify mechanisms and guide treatment development. Future research should explore psychodynamics integration into chronic pain care and its impact on long-term outcomes. The mission is clear: we build connection with the patient—and in that shared space, nurture the threads that reconnect them to their self, their body, and the world beyond.
